# Integrative analysis of PANoptosis-related genes in diabetic retinopathy: machine learning identification and experimental validation

**DOI:** 10.3389/fimmu.2024.1486251

**Published:** 2024-12-04

**Authors:** Han Chen, Enguang Chen, Ting Cao, Feifan Feng, Min Lin, Xuan Wang, Yu Xu

**Affiliations:** ^1^ Department of Ophthalmology, Xinhua Hospital, School of Medicine, Shanghai Jiao Tong University, Shanghai, China; ^2^ Shanghai Jiao Tong University School of Medicine, Shanghai, China

**Keywords:** diabetic retinopathy, PANoptosis, machine learning, bioinformatics analysis, differentially expressed genes, biomarkers

## Abstract

**Background:**

Diabetic retinopathy (DR) is a major complication of diabetes, leading to severe vision impairment. Understanding the molecular mechanisms, particularly PANoptosis, underlying DR is crucial for identifying potential biomarkers and therapeutic targets. This study aims to identify differentially expressed PANoptosis-related genes (DE-PRGs) in DR, offering insights into the disease’s pathogenesis and potential diagnostic tools.

**Methods:**

DR datasets were obtained from the Gene Expression Omnibus (GEO) database, while PANoptosis-related genes were sourced from the GeneCards database. Differentially expressed genes (DEGs) were identified using the DESeq2 package, followed by functional enrichment analysis through DAVID and Metascape tools. Three machine learning algorithms—LASSO regression, Random Forest, and SVM-RFE—were employed to identify hub genes. A diagnostic nomogram was constructed and its performance assessed via ROC analysis. The CIBERSORT algorithm analyzed immune cell infiltration. Hub genes were validated through RT-qPCR, Western blotting, immunohistochemistry, and publicly available datasets. Additionally, the impact of FASN and PLSCR3 knockdown on HUVECs behavior was validated through *in vitro* experiments.

**Results:**

Differential expression analysis identified 1,418 DEGs in the GSE221521 dataset, with 39 overlapping DE-PRGs (29 upregulated, 10 downregulated). Functional enrichment indicated that DE-PRGs are involved in apoptosis, signal transduction, and inflammatory responses, with key pathways such as MAPK and TNF signaling. Machine learning algorithms identified six PANoptosis-related hub genes (BEX2, CASP2, CD36, FASN, OSMR, and PLSCR3) as potential biomarkers. A diagnostic nomogram based on these hub genes showed high diagnostic accuracy. Immune cell infiltration analysis revealed significant differences in immune cell patterns between control and DR groups, especially in Activated CD4 Memory T Cells and Monocytes. Validation confirmed the diagnostic efficiency and expression patterns of the PANoptosis-related hub genes, supported by *in vitro* and the GSE60436 dataset analysis. Furthermore, experiments demonstrated that knocking down FASN and PLSCR3 impacted HUVECs behavior.

**Conclusion:**

This study provides valuable insights into the molecular mechanisms of DR, particularly highlighting PANoptosis-related pathways, and identifies potential biomarkers and therapeutic targets for the disease.

## 1 Introduction

Diabetic retinopathy (DR) is one of the most common microvascular complications of diabetes mellitus and a leading cause of vision impairment and blindness among working-age adults worldwide (1, 2). The increasing prevalence of diabetes has contributed to a corresponding rise in the incidence of DR, making it a significant public health concern (3). DR is characterized by damage to the retinal microvasculature, leading to a series of pathological changes including microaneurysms, hemorrhages, retinal edema, and neovascularization (4, 5).

The pathogenesis of DR is multifactorial and complex, involving a combination of genetic, metabolic, and environmental factors (6). Hyperglycemia-induced oxidative stress, inflammation, and dysregulation of angiogenic factors play pivotal roles in the progression of the disease (7–9). Recent advances in understanding the molecular mechanisms of DR have highlighted the importance of various cell death pathways, including apoptosis, necroptosis, and pyroptosis, collectively referred to as PANoptosis, in retinal cell injury and vascular dysfunction (10, 11).

Despite significant progress in elucidating the mechanisms underlying DR, there remains a critical need for the identification of specific molecular targets that can be leveraged for diagnostic and therapeutic purposes (12). Effective treatment strategies have been hampered by the heterogeneity of the disease and the lack of reliable biomarkers for early detection and progression monitoring. In this context, the identification of differentially expressed PANoptosis-related genes (DE-PRGs) holds promise for advancing our understanding of DR and discovering novel therapeutic targets.

In our study, we aimed to address the challenges in DR by identifying DE-PRGs and exploring their potential as biomarkers and therapeutic targets. Using high-throughput sequencing data from the Gene Expression Omnibus (GEO) database, we identified 1418 differentially expressed genes (DEGs) in the GSE221521 dataset, with 39 specifically related to PANoptosis. Functional enrichment analysis revealed these DE-PRGs are key players in apoptosis regulation, signal transduction, and inflammatory responses, with involvement in pathways such as MAPK and TNF signaling. By employing machine learning algorithms such as LASSO regression, Random Forest, and Support Vector Machine - Recursive Feature Elimination (SVM-RFE), we identified six hub genes (BEX2, CASP2, CD36, FASN, OSMR, and PLSCR3), which were validated via RT-qPCR and immunohistochemistry (IHC) in both *in vitro* models and clinical samples. A diagnostic nomogram based on these hub genes demonstrated high predictive capability, enhancing clinical decision-making. Additionally, Gene Set Variation Analysis (GSVA) and immune cell infiltration analysis further underscored the relevance of these hub genes in DR pathology and highlighted their potential as novel therapeutic targets. Our findings provide a foundation for future research into personalized medicine approaches for the effective management and treatment of DR.

## 2 Methods

### 2.1 Data collection

Microarray data of the GSE60436 dataset and high-throughput sequencing data of the GSE221521 dataset were downloaded from the GEO database (https://www.ncbi.nlm.nih.gov/geo/) (13). The GSE221521 dataset comprised gene expression profiling analysis of RNA-seq data, involving blood samples from 50 healthy controls and 69 individuals with DR. The GSE60436 dataset contained microarray data on gene expression profiles of fibrovascular membranes, including samples from 6 individuals with proliferative diabetic retinopathy (PDR) and 3 healthy controls. The GSE221521 dataset was designated as the training set for identifying DEGs, while the GSE60436 dataset was used as the validation set. PANoptosis-related genes were identified through a search in the GeneCards database, based on a correlation score greater than 3.

### 2.2 Identification of differentially expressed PANoptosis-related genes

Differential gene expression analysis was conducted separately for each dataset. For the GSE221521 dataset, the DESeq2 package was used to normalize raw read counts and identify DEGs by comparing DR samples with healthy controls (14). Genes with |log2 Fold Change| > 0.585 and p-value < 0.05 were considered significant DEGs. In this study, we identified the overlapping genes between DEGs and PANoptosis-related genes, defining them as DE-PRGs for subsequent analysis.

### 2.3 GO and KEGG pathway enrichment analysis

To explore the functional and interactive roles of these PANoptosis-related DEGs in biological pathways, we utilized the DAVID online database to perform GO and KEGG pathway enrichment analyses (15). A significance level of p-value < 0.05 was used as the threshold criterion. In this study, the results were visualized using the bioinformatics platform (https://www.bioinformatics.com.cn). Additionally, the GO enrichment analysis was supplemented using the Metascape online tool (https://metascape.org) (16).

### 2.4 Gene set variation analysis

We also quantified the activity of 50 hallmark pathways using the GSVA R package to uncover the potential biological functions of key genes (17). Gene sets were obtained from the Molecular Signatures Database (MSigDB) (18). In this analysis, significance criteria of |t| > 1 and p < 0.05 were applied to select significant differences, where t > 1 and t < -1 represent pathway activation in the high-expression and low-expression groups, respectively.

### 2.5 Machine learning

To identify candidate biomarkers and establish a diagnostic model, we employed three machine learning algorithms: LASSO regression, Random Forest, and SVM-RFE (19, 20). The genes identified at the intersection of these three algorithms were considered as the hub genes for diagnosis.

### 2.6 Nomogram construction and receiver operating characteristic evaluation

The hub genes were used to construct a nomogram with the “rms” R package, facilitating the prediction of DR risk. Receiver Operating Characteristic (ROC) curve analysis was conducted to assess the diagnostic performance of the hub genes and the nomogram, with the Area Under the Curve (AUC) value providing a measure of diagnostic accuracy.

### 2.7 Analysis of immune cell infiltration

We estimated the relative abundance of immune-infiltrating cells using the CIBERSORT algorithm (21). Bar and box plots were generated to illustrate immune cell proportions and their differences between healthy controls and the DR group. The “ggplot2” package was utilized to visualize the correlations between hub genes and immune cell types (22).

### 2.8 Cell culture and RNA extraction

Human umbilical vein endothelial cells (HUVECs) (ATCC, Cat. CRL-1730) were purchased from ATCC and cultured in endothelial cell medium supplemented with 10% fetal bovine serum (FBS) and 1% penicillin-streptomycin at 37°C in a 5% CO2 incubator. To establish a DR cell model, cells were treated with high glucose (30 mM) for 48 hours, while the control group was treated with normal glucose (5 mM) for the same duration. RNA was extracted from HUVECs using the RNA Quick Purification Kit (SB-C6015, Share-bio, Shang Hai).

### 2.9 Clinical sample collection and RNA extraction

Samples were obtained from DR patients and healthy controls at the ophthalmology department of Xinhua Hospital, with informed consent and Institutional Review Board approval from Xin Hua Hospital Affiliated to Shanghai Jiao Tong University School of Medicine (Approval No. XHEC-D-2024-146). Peripheral blood was collected in EDTA-coated tubes and processed within two hours. Total RNA was extracted using the EZ-press Whole Blood RNA Purification Kit (B0006, EZBioscience).

### 2.10 Quantitative real-time and western blotting

To assess mRNA expression levels, cDNA synthesis was performed on extracted RNA using the reverse transcription kit (RR036A, Takara). RT-qPCR was conducted with the TB Green^®^ Premix Ex Taq™ II kit (RR820A, Takara). Primer sequences for the core genes are provided in **Supplementary Table S1**. β-actin served as the internal control. Relative expression levels were calculated using the 2^^-ΔΔCt^ method.

Protein expression was analyzed by Western blotting. Cells were lysed, and proteins were separated by SDS-PAGE before being transferred to PVDF membranes. Membranes were blocked and then incubated with primary antibodies: CD36 (#28109, Cell Signaling), FASN (A19050, Abclonal), PLSCR3 (A20915, Abclonal), and β-actin (SB-AB2001, Share-bio) as a loading control. After washing, membranes were treated with an HRP-conjugated secondary anti-rabbit IgG antibody (SA00001-2, Proteintech). Bands were detected using an enhanced chemiluminescence system.

### 2.11 Immunohistochemistry staining

Fibrovascular membrane samples in patients with PDR were fixed in formalin for 24 hours, paraffin-embedded, and sectioned at a thickness of 4 µm for IHC staining. Paraffin-embedded tissue sections underwent deparaffinization using xylene and rehydration through graded ethanol solutions to distilled water. Antigen retrieval was performed by heating the sections at 94°C for 25 minutes in a citrate buffer (pH 6.0), followed by cooling to room temperature. To block endogenous peroxidase activity, sections were incubated in a 3% hydrogen peroxide solution at room temperature for 25 minutes, protected from light. Non-specific binding was prevented by blocking with 3% bovine serum albumin (BSA) for 30 minutes at room temperature. The sections were then incubated overnight at 4°C with primary antibodies, including Anti-CD36 (1:600, GB112562-100, Servicebio), Anti-FASN (1:200, GB15546-100, Servicebio) and Anti-PLSCR3 (1:100, GB11859-100, Servicebio). After primary antibody incubation, secondary antibodies were applied, and sections were developed with DAB substrate. The sections were counterstained with hematoxylin, and images were captured using the Zeiss fluorescence microscope.

### 2.12 *In vitro* experiments

Cell transfection was performed using Lipofectamine™ 3000 reagent (L3000015, Invitrogen). Following the manufacturer’s instructions, small-interfering RNA (siRNA) was mixed with the transfection reagent and added to the cell culture medium. The specific siRNA sequences used in the experiments are listed in **Supplementary Table S2**.

Cell proliferation was assessed using the 5-ethynyl-2’-deoxyuridine (EdU) incorporation assay. The Click-iT EdU Imaging Kit (SB-C6015, Share-bio, Shang Hai) was used following the manufacturer’s protocol. EdU-positive cells were observed and counted under a fluorescence microscope. The percentage of EdU-positive cells was calculated using the formula: EdU-positive cell percentage (%) = [count of EdU-positive cells (green)/count of Hoechst-33342-stained cells (blue)] × 100%.

Cell viability was measured using the Cell Counting Kit-8 (SB-CCK8, Share-bio, Shang Hai). Cells were seeded in a 96-well plate, and CCK-8 reagent was added followed by incubation at 37°C for 1 hours. Absorbance was measured at 450 nm using a microplate reader to evaluate cell proliferation.

The wound healing assay was used to assess cell migration ability. Cells were seeded in a 6-well plate and allowed to reach 90% confluence. A straight line was scratched across the cell monolayer using a sterile pipette tip. Images of the scratch area were captured at 0 hours and 36 hours using a microscope to evaluate cell migration. The wound closure percentage was calculated using the formula: Wound closure rate (%) = [(initial wound area - remaining wound area)/initial wound area] × 100%.

### 2.13 Statistical analysis

For group comparisons, an independent Student’s t-test was used to analyze variables with a normal distribution, while the Wilcoxon rank-sum test was employed for those without a normal distribution. Statistical analyses were performed using R (version 4.2.0), with a significance threshold set at p < 0.05.

## 3 Results

### 3.1 Identification of differentially expressed PANoptosis-related genes

**Figure 1:**
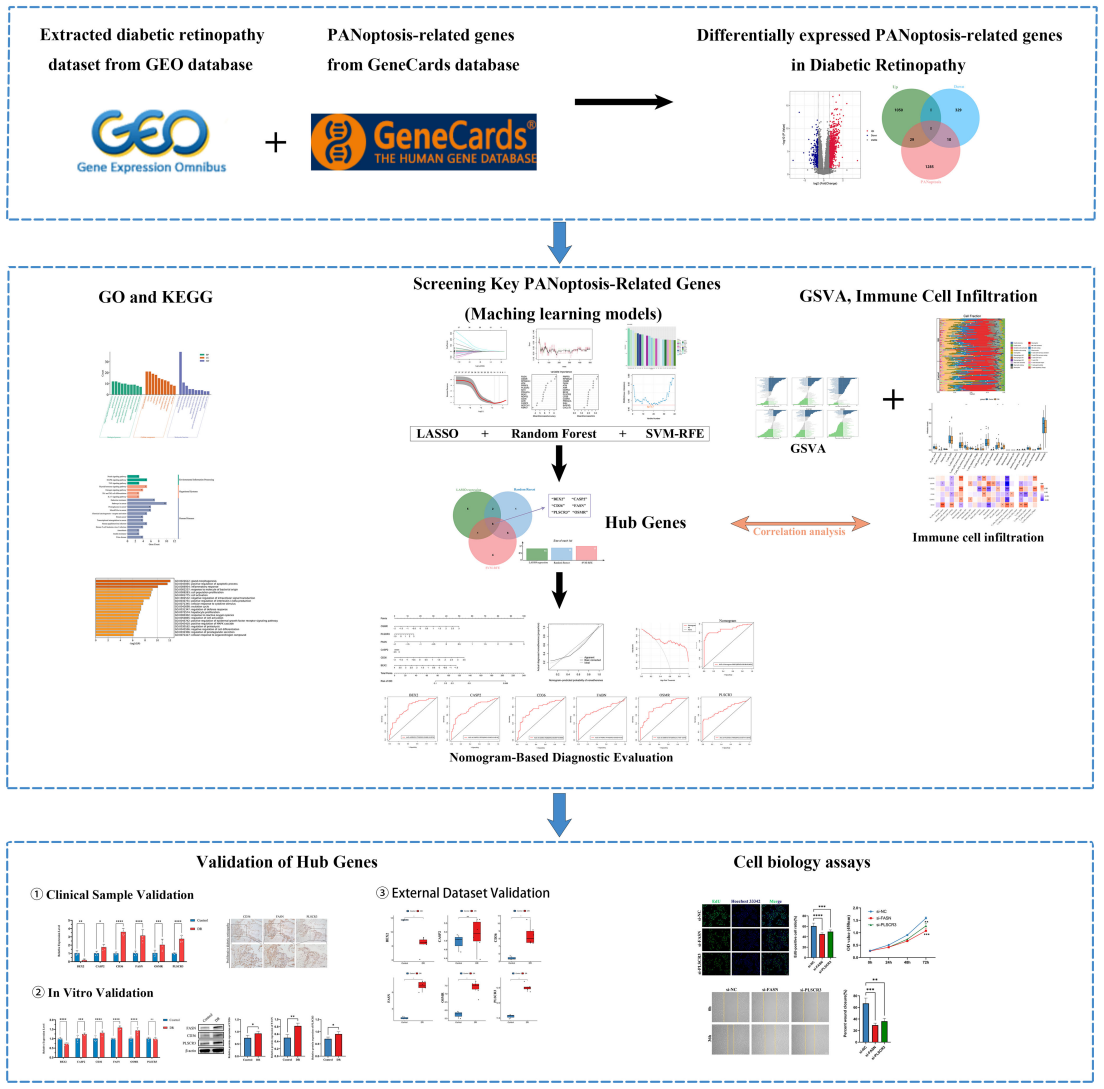
Flowchart of the research. (ns indicates no significance, *p < 0.05, **p < 0.01, ***p < 0.005, ****p < 0.0001).

**Figure 2:**
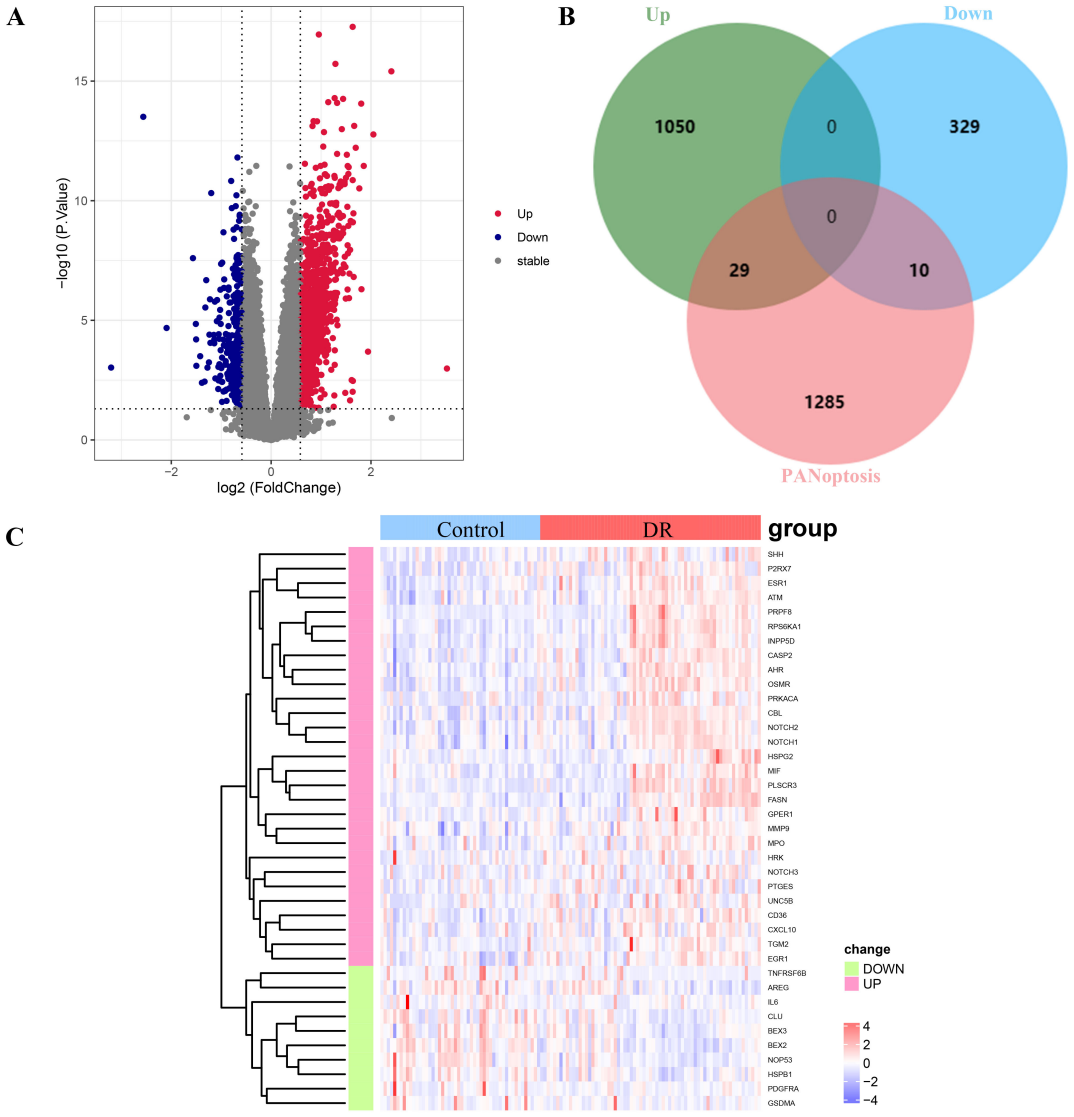
Analysis of differentially expressed PANoptosis-related genes (DE-PRGs) in DR. **(A)** Volcano plot showing DEGs in the GSE221521 dataset. Red dots indicate upregulated genes, blue dots indicate downregulated genes, and gray dots indicate non-significant genes. **(B)** Venn diagram illustrating the overlap between DEGs and PANoptosis-related genes from the GeneCards database. A total of 39 DE-PRGs were identified (29 upregulated, 10 downregulated). **(C)** Heatmap displaying the expression levels of the 39 overlapping DE-PRGs in the GSE221521 dataset. Red indicates upregulation, and blue indicates downregulation.

The overall design is shown in **Figure 1**. A total of 1418 DEGs were identified in the GSE221521 dataset, with 1079 genes significantly upregulated and 339 genes significantly downregulated. The volcano plot of DEGs is shown in **Figure 2A**. Using a correlation score threshold of >3, we identified 1324 PANoptosis-related genes from the GeneCards database, comprising 1313 apoptosis genes, 11 necrosis genes, and 31 pyroptosis genes (**Supplementary Table S3**). Through Venn diagram analysis, we identified 39 overlapping genes, of which 29 were upregulated and 10 were downregulated as DE-PRGs (**Figures 2B, C**).

### 3.2 Functional enrichment analysis

**Figure 3:**
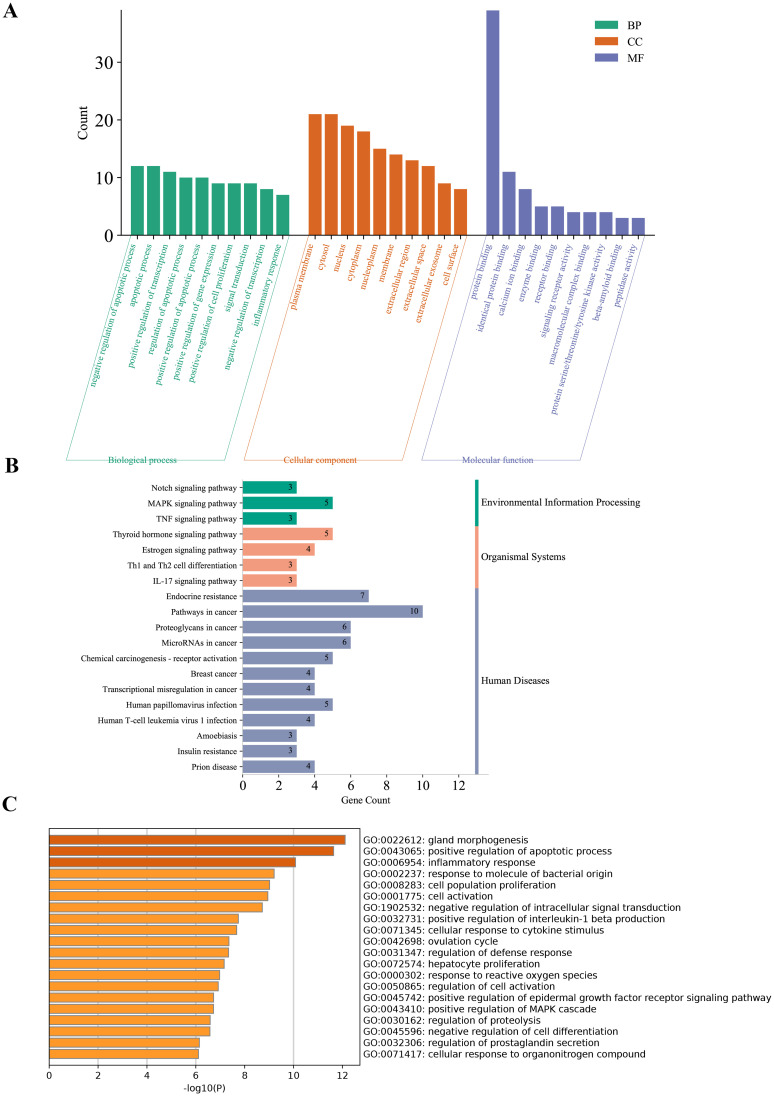
Functional enrichment analysis of DE-PRGs in DR. **(A)** GO enrichment analysis of DE-PRGs. The bar plot shows the top enriched GO terms across three categories: Biological Processes (BP), Cellular Components (CC), and Molecular Functions (MF). **(B)** KEGG pathway analysis of DE-PRGs. **(C)** Supplementary GO enrichment analysis performed using the Metascape online tool.

Functional enrichment analysis revealed that the DE-PRGs in DR were significantly involved in several key biological processes and pathways. GO analysis indicated that these genes play crucial roles in the regulation of apoptotic processes, signal transduction, and inflammatory responses (**Figure 3A**). They were predominantly associated with cellular components such as the plasma membrane, cytosol, nucleus, and extracellular regions, and were enriched in molecular functions including protein binding, enzyme binding, and receptor binding activities.

KEGG pathway analysis revealed that these genes are involved in critical DR-related pathways, including endocrine resistance, thyroid hormone signaling, estrogen signaling, MAPK signaling, and the TNF signaling pathway, highlighting their roles in inflammation, cellular stress responses, and metabolic dysregulation (**Figure 3B**). 

Supplementary GO enrichment analysis using the Metascape tool further confirmed the involvement of DE-PRGs in pathways related to endocrine resistance, inflammation, and signaling mechanisms crucial for retinal health and disease (**Figure 3C**).

### 3.3 Screening key PANoptosis-related genes via machine learning

**Figure 4:**
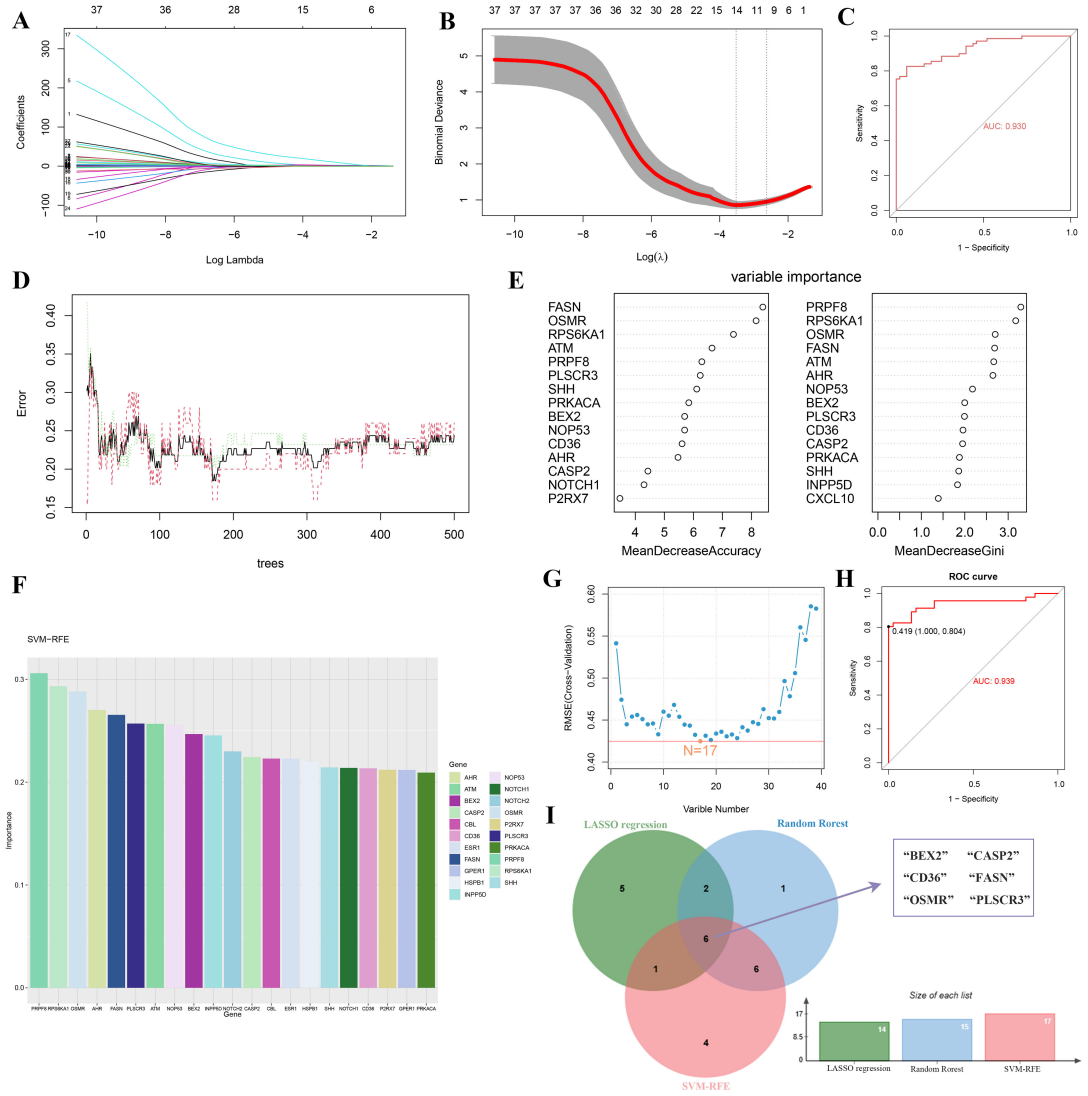
Identification of key hub genes for PANoptosis-related genes in DR using machine learning algorithms. **(A-C)** LASSO regression analysis: **(A)** Coefficient profiles of the 14 potential hub genes, **(B)** Tuning parameter (lambda) selection in the LASSO model using 10-fold cross-validation, and **(C)** ROC curve with an AUC of 0.93. **(D, E)** Random Forest (RF) algorithm: **(D)** Error rate of the RF model as a function of the number of trees, and **(E)** Variable importance plot ranking the genes based on their importance. **(F-H)** Support Vector Machine-Recursive Feature Elimination (SVM-RFE) method: **(F)** Bar plot of the 17 candidate biomarkers, **(G)** Cross-validation error curve for the SVM-RFE model, and **(H)** ROC curve with an AUC of 0.939. **(I)** Venn diagram showing the intersection of hub genes identified by RF, RF, and SVM-RFE methods, resulting in six key hub genes (BEX2, CASP2, CD36, FASN, OSMR, and PLSCR3).

Using LASSO regression, RF algorithm, and SVM-RFE, we screened potential candidate hub genes for DE-PRGs. LASSO regression identified 14 potential hub genes and evaluated model performance using ROC curves, with an AUC of 0.93 (**Figures 4A–C**). The RF algorithm ranked these genes based on their importance (**Figures 4D, E**). The SVM-RFE method extracted 17 genes as candidate biomarkers and similarly evaluated model performance using ROC curves, with an AUC of 0.939 (**Figures 4F–H**). By intersecting the results from these three methods, we identified six key hub genes (BEX2, CASP2, CD36, FASN, OSMR, and PLSCR3) as potential biomarkers for DR (**Figure 4I**).

### 3.4 Nomogram-based diagnostic evaluation

**Figure 5:**
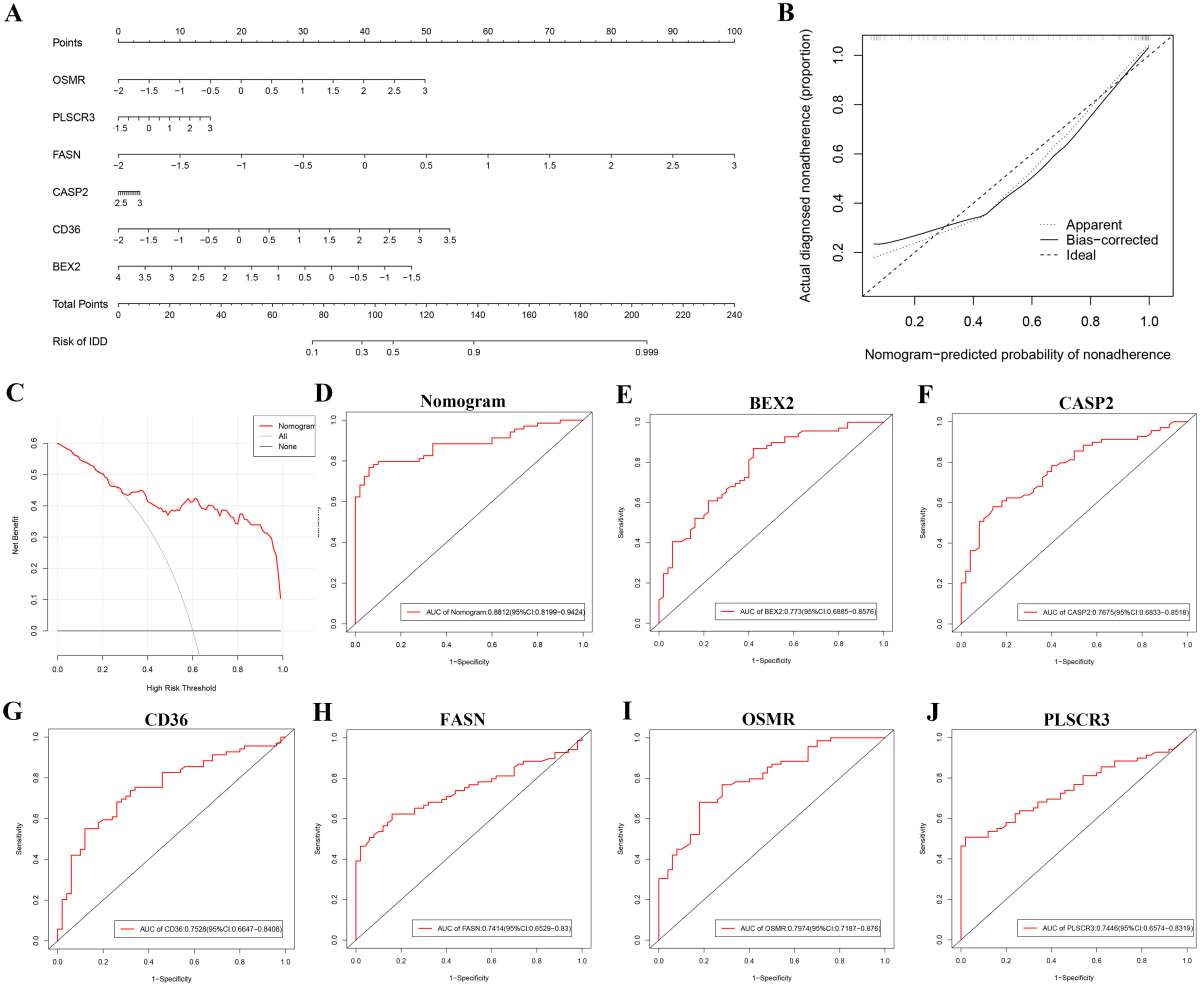
Nomogram-based diagnostic evaluation for DR using six identified hub genes. **(A)** Nomogram developed for predicting the probability of DR based on the six hub genes (BEX2, CASP2, CD36, FASN, OSMR, and PLSCR3). **(B)** Calibration curve of the nomogram, showing the agreement between predicted probabilities and actual observed outcomes. **(C)** Decision curve analysis (DCA) demonstrating the clinical utility of the nomogram for DR diagnosis. **(D-J)** ROC curves for the nomogram and each of the six hub genes: **(D)** Nomogram, **(E)** BEX2, **(F)** CASP2, **(G)** CD36, **(H)** FASN, **(I)** OSMR, and **(J)** PLSCR3.

To enhance the diagnosis and prediction capabilities for the six identified hub genes, we developed a nomogram (**Figure 5A**). The calibration curve indicated a high agreement between the predicted probabilities from our nomogram diagnostic model and the actual observed outcomes (**Figure 5B**). Moreover, decision curve analysis (DCA) highlighted the potential benefits of using the nomogram for clinical decision-making in the diagnosis of DR (**Figure 5C**). The calculated AUCs and their 95% confidence intervals for the nomogram as well as for each hub gene are provided (**Figures 5D–J**). Notably, all six hub genes achieved AUC values exceeding 0.7, and the nomogram demonstrated an even higher AUC compared to any single hub gene, underscoring its robust diagnostic capability for DR.

### 3.5 GSVA analysis of six hub genes in DR

**Figure 6:**
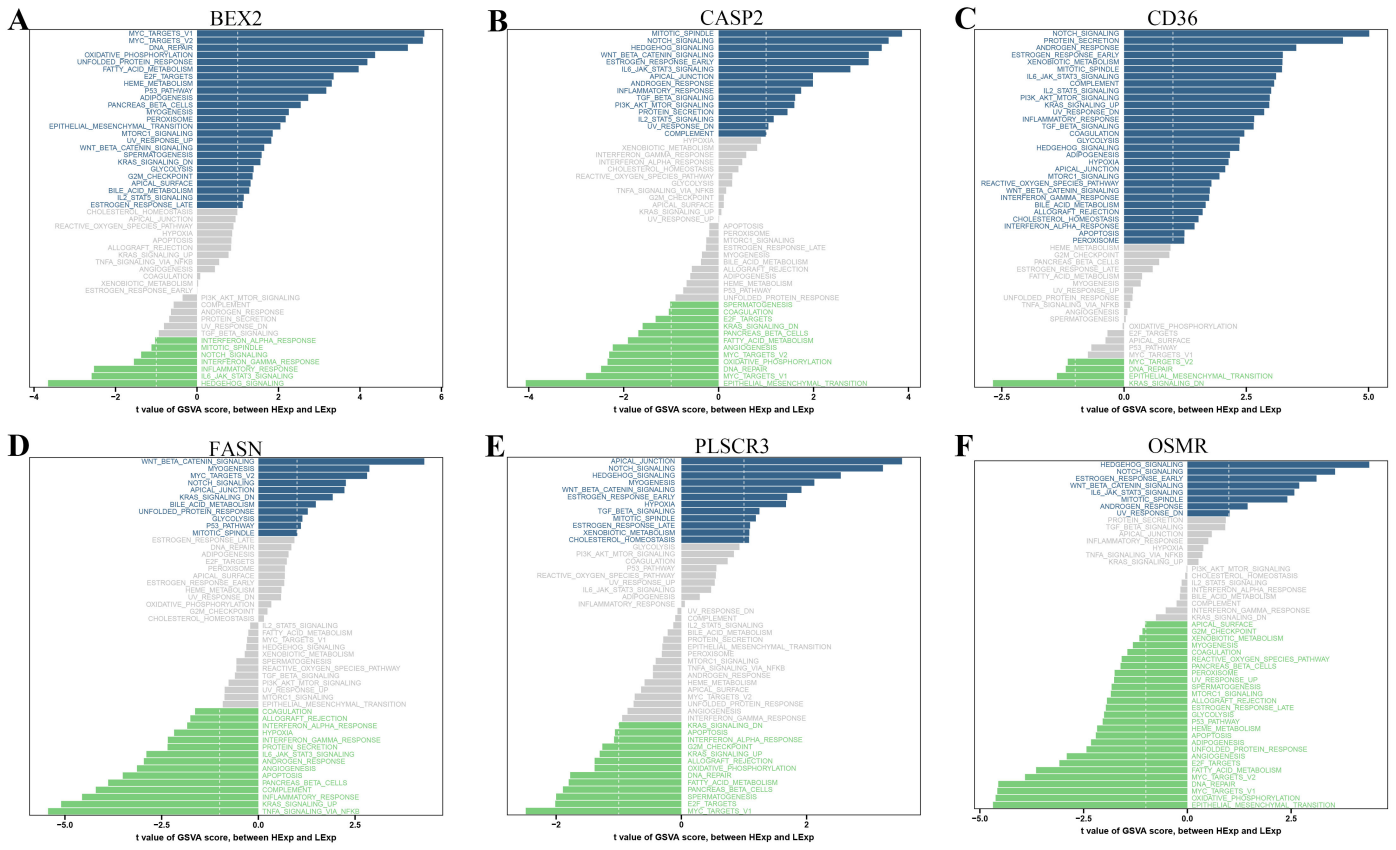
Gene Set Variation Analysis (GSVA) of PANoptosis-related genes. **(A-F)** GSVA results showing the correlation between specific PANoptosis-related genes and various signaling pathways: **(A)** BEX2, **(B)** CASP2, **(C)** CD36, **(D)** FASN, **(E)** PLSCR3, and **(F)** OSMR. The bar plots display the t-values of GSVA scores between DR and control samples.

Our GSVA analysis revealed significant correlations between specific genes and signaling pathways (**Figure 6**). Notably, CD36, CASP2, FASN, OSMR, and PLSCR3 showed a significant positive correlation with the “WNT_BETA_CATENIN_SIGNALING” pathway and a significant negative correlation with the “NOTCH_SIGNALING” pathway. In contrast, BEX2 exhibited an opposite trend, being negatively correlated with “WNT_BETA_CATENIN_SIGNALING” and positively correlated with “NOTCH_SIGNALING”. Additionally, we observed that “APOPTOSIS” was significantly positively correlated with CD36.

### 3.6 Immune cell infiltration analysis

**Figure 7:**
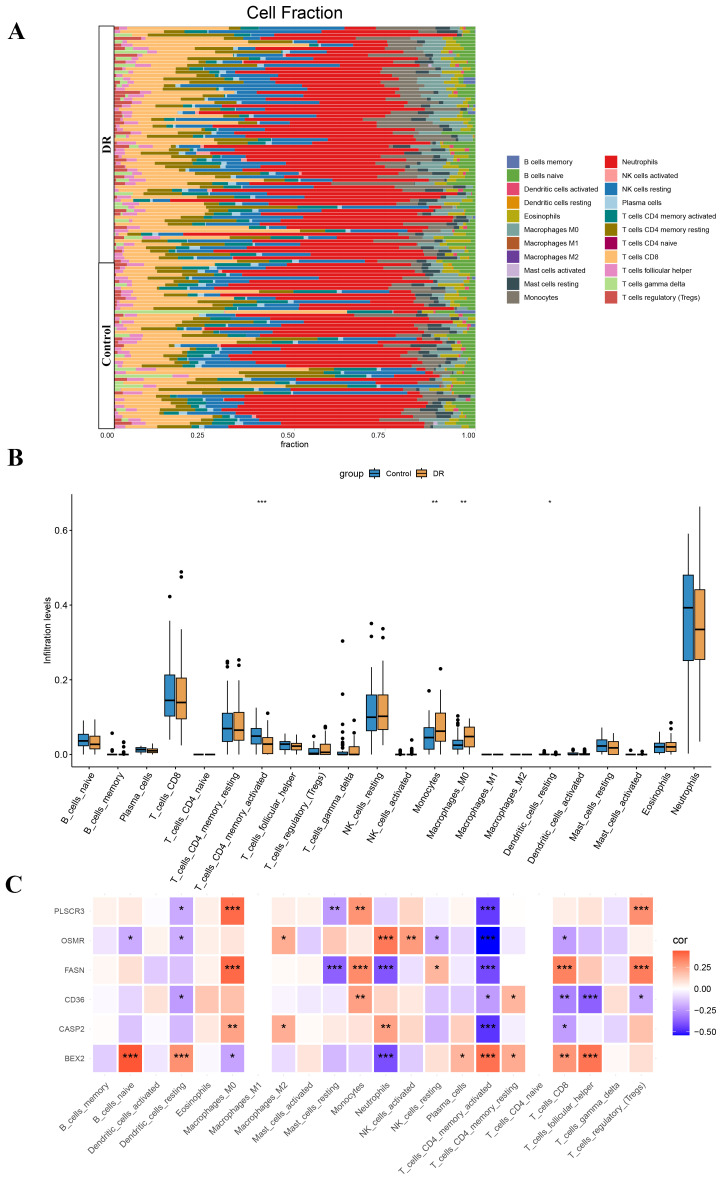
Immune cell infiltration analysis in diabetic retinopathy (DR). **(A)** Stacked bar plot showing the proportion of 22 types of immune cells in DR and control samples. **(B)** Box plot illustrating the significant differences in the proportions of Activated CD4 Memory T Cells, Monocytes, and M0 Macrophages between DR and control samples. Activated CD4 Memory T Cells were significantly downregulated in DR patients, while Monocytes and M0 Macrophages were significantly upregulated. **(C)** Heatmap displaying the correlation between the six hub genes (BEX2, CASP2, CD36, FASN, OSMR, and PLSCR3) and the 22 types of immune cells. *p < 0.05, **p < 0.01, ***p < 0.001.

Our findings suggest that DE-PRGs are significantly associated with immune cell infiltration in DR. Therefore, we proceeded with immune cell infiltration analysis to gain further insights into the involvement of the immune system in DR (**Figure 7A**). From the dataset of DE-PRGs, we identified that Activated CD4 Memory T Cells, Monocytes, and M0 Macrophages exhibited significant differences. Notably, in DR patients, Activated CD4 Memory T Cells were significantly downregulated, while monocytes and M0 Macrophages were significantly upregulated (**Figure 7B**). Furthermore, we conducted a correlation analysis between the six hub genes and the 22 types of immune cells. The analysis revealed that Activated CD4 Memory T Cells had a significant negative correlation with BEX2, while showing significant positive correlations with PLSCR3, OSMR, FASN, CD36, and CASP2 (**Figure 7C**). These findings indicated that various immune cell types infiltrate differently in DR patients, highlighting potential targets for novel therapeutic strategies.

### 3.7 Validation of PANoptosis-related hub genes

**Figure 8:**
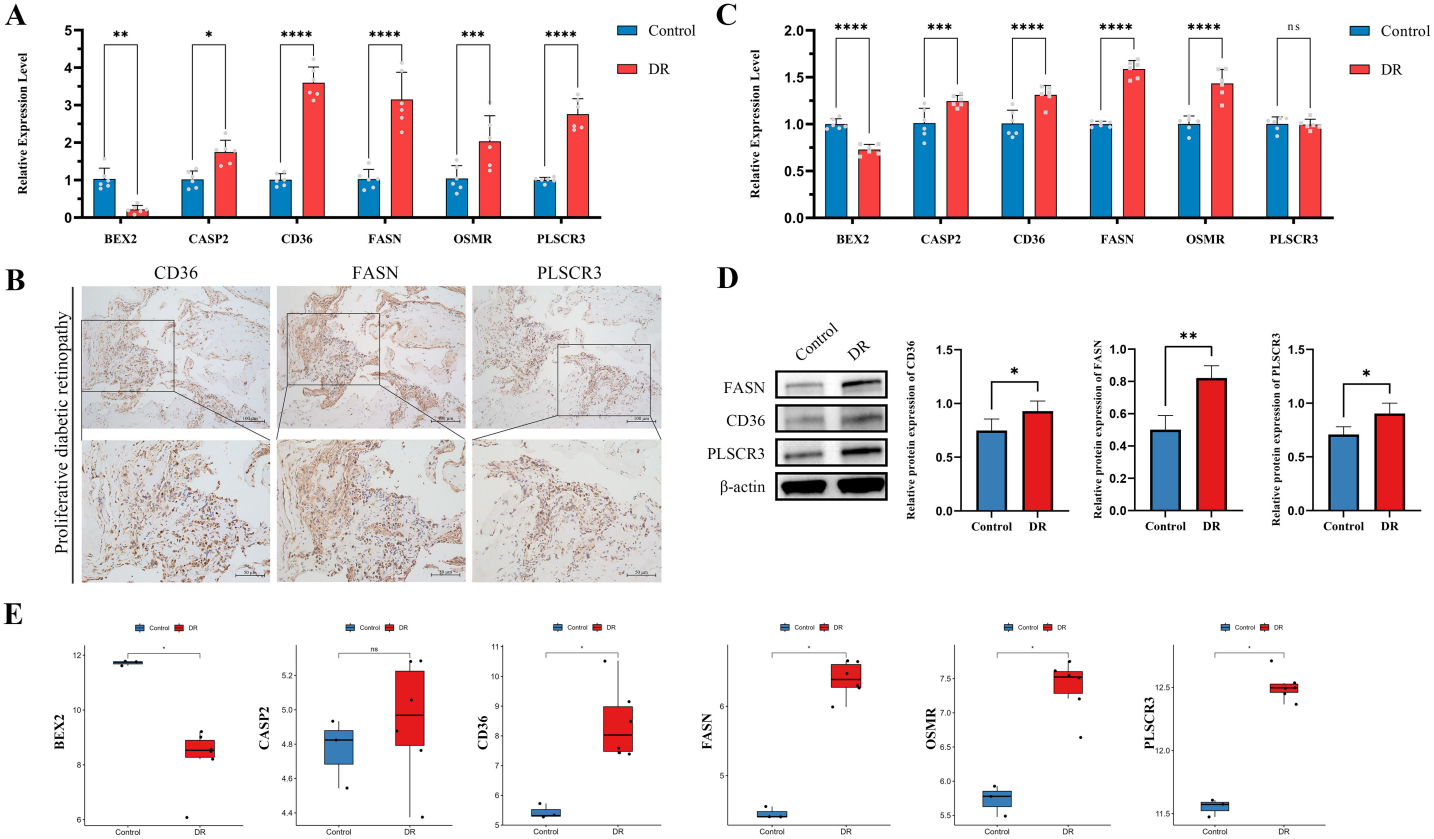
Validation of six hub genes in diabetic retinopathy (DR) through multiple approaches. **(A)** RT-qPCR validation of six hub genes in clinical blood samples from DR patients compared to healthy controls. **(B)** IHC validation in proliferative membrane samples from PDR patients. The images show the protein expression levels of CD36, FASN and PLSCR3 in the proliferative membranes (Scale bar: 100 μm or 50 μm). **(C)** RT-qPCR validation of six hub genes in HUVECs exposed to high glucose conditions compared to controls. **(D)** The protein levels of CD36, FASN, and PLSCR3 in HUVECs were evaluated by western blot after exposure to high glucose conditions compared to controls. **(E)** Validation using public dataset GSE60436. (ns indicates no significance, *p < 0.05, **p < 0.01, ***p < 0.005, ****p < 0.0001).

To comprehensively validate the expression and roles of the six identified hub genes (BEX2, CASP2, CD36, FASN, OSMR, and PLSCR3), we employed multiple methodologies. Initially, RT-qPCR was performed on blood samples from clinical patients. Results consistently showed that, compared to healthy controls, DR patients exhibited significantly higher expression levels of CASP2, CD36, FASN, OSMR, and PLSCR3, while BEX2 was significantly downregulated (**Figure 8A**). Furthermore, IHC staining was conducted on proliferative fibrovascular membranes from patients with PDR to validate the protein levels of CD36, FASN, and PLSCR3. The findings revealed high expression of these genes in fibrovascular membranes, aligning with the transcriptional data from RT-qPCR (**Figure 8B**).

In the second phase, an *in vitro* DR cell model was utilized to assess the expression levels of the six target genes. Notably, in HUVECs exposed to high glucose conditions, all hub genes except PLSCR3 showed expression patterns that mirrored our previous findings (**Figure 8C**). Western blotting was also performed to validate the protein expression levels of CD36, FASN, and PLSCR3. The results corroborated the transcriptional alterations, confirming upregulation at the protein level (**Figure 8D**).

Additionally, findings were further validated using the public dataset GSE60436, which includes RNA sequencing data from the fibrovascular membranes of PDR patients. Analysis showed that the expression patterns of the six hub genes in this dataset were consistent with observations from RT-qPCR and protein expression validations. This consistency underscored the robustness and universality of these hub genes (**Figure 8E**).

### 3.8 PANoptosis-related hub genes promoted proliferation and migration in HUVECs

**Figure 9:**
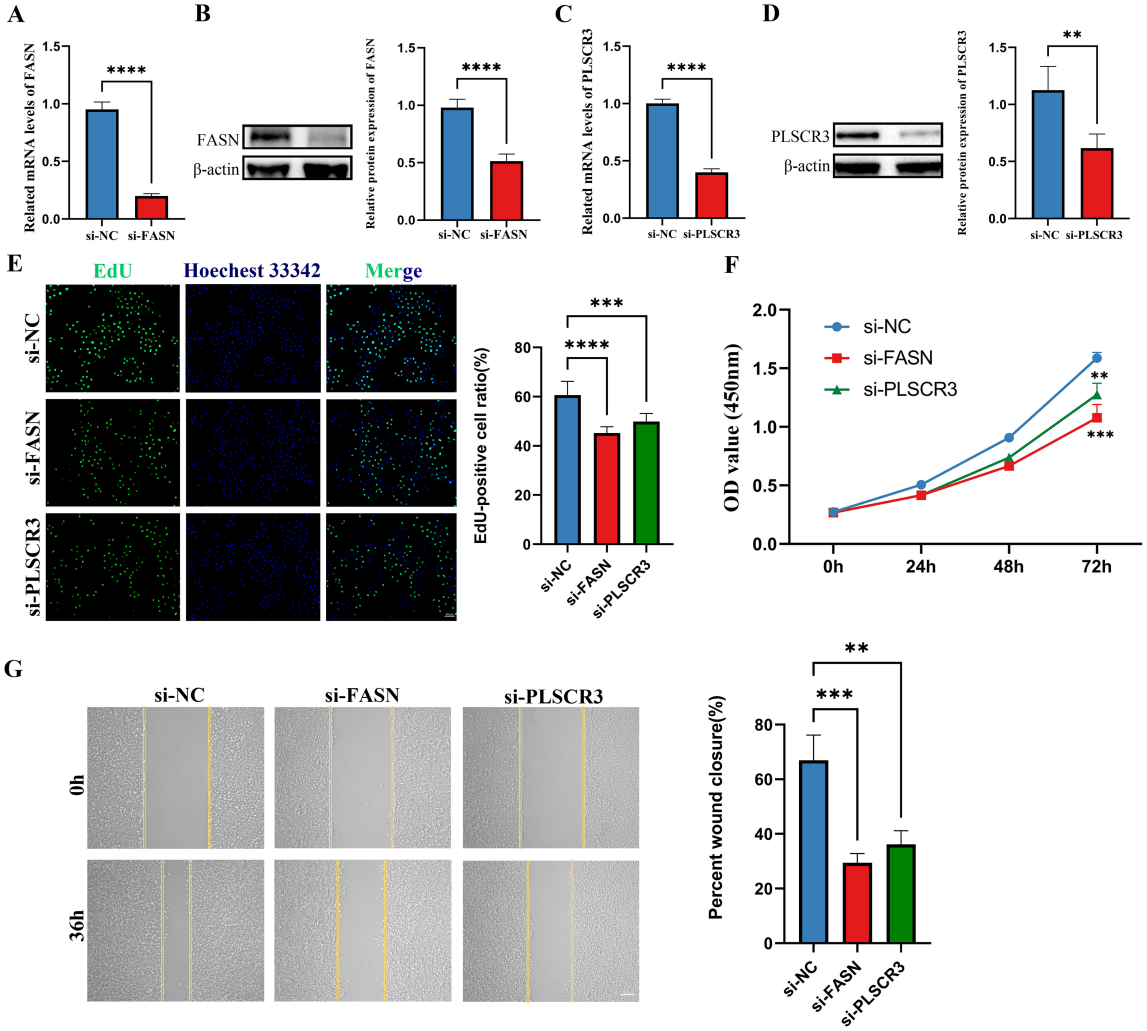
Knockdown of FASN and PLSCR3 inhibits HUVECs proliferation and migration. **(A, B)** The knockdown efficiency of FASN were detected by RT-qPCR and western-blot in HUVECs. **(C, D)** The knockdown efficiency of PLSCR3 were detected by RT-qPCR and western-blot in HUVECs. **(E)** EdU assays were performed on HUVECs transfected with negative control (siNC), siRNA-FASN, and siRNA-PLSCR3 to assess cell proliferation (Scale bar: 50 μm). **(F)** CCK8 assays were performed on HUVECs transfected with negative control, siRNA-FASN, and siRNA-PLSCR3 to assess cell proliferation. **(G)** Cell migration was assessed with a wound-healing assay. Representative images and wound closure analysis at 0 and 36 h are shown (Scale bar: 200 μm). **p < 0.01; ***p < 0.001; ****p < 0.0001.

Following the validation of gene expression, we proceeded with *in vitro* cell experiments, utilizing siRNA to knock down the genes CD36, FASN, and PLSCR3 in HUVECs. The efficiency of the knockdown was assessed using Western blotting and RT-qPCR. Results showed that FASN and PLSCR3 were significantly downregulated at both the mRNA and protein levels (**Figures 9A–D**). In contrast, the knockdown of CD36 was less effective (**Supplementary Figure S1**).

Biological assays further revealed that silencing FASN or PLSCR3 significantly inhibited the proliferation (**Figures 9E, F**) and migration (**Figure 9G**) of HUVECs. These findings suggest that FASN and PLSCR3 play crucial roles in promoting cell proliferation and migration, potentially contributing to the progression of diabetic retinopathy.

## 4 Discussion

DR ranks among the top causes of blindness in adults globally, posing a significant public health challenge (23, 24). PANoptosis, a unique form of programmed cell death that integrates the pathways of pyroptosis, apoptosis, and necroptosis, has garnered attention due to its role in inflammatory responses and cellular homeostasis (25, 26). Although the individual pathways of pyroptosis, apoptosis, and necroptosis are well-studied, the interplay and regulation among them in PANoptosis remain complex and not fully understood. Recent research in the context of glaucomatous retinal ganglion cell (RGC) damage has highlighted the involvement of PANoptosis. For instance, treatment with melatonin has been shown to rescue RGC survival and reduce the loss of retinal nerve fiber layer thickness, potentially by inhibiting the expression of PANoptosis-associated proteins (26). Our study aimed to elucidate the role of PANoptosis-related genes in DR and leverage machine learning techniques to identify and validate key genetic biomarkers, providing a comprehensive understanding of the underlying mechanisms and potential therapeutic targets.

In our analysis, we identified 1418 DEGs from the GSE221521 dataset, of which 39 were PANoptosis-related after intersecting with a curated list of 1324 PANoptosis-related genes. Functional enrichment analysis revealed that these genes are implicated in crucial biological processes such as apoptotic regulation, cytokine production, immune responses, and response to chemical stimuli. Pathway analysis pinpointed significant involvement in TNF signaling, Toll-like receptor signaling, MAPK pathway, and other critical inflammation-related pathways. These findings highlighted the integral role of PANoptosis in mediating inflammatory and immune responses in DR.

The pathogenesis of DR involves hyperglycemia-induced damage, which in turn leads to chronic inflammation and subsequent retinal damage (27–30). Chronic hyperglycemia in DR induces oxidative stress and inflammatory cytokine production, activating PANoptosis pathways (31, 32). This activation results in the release of pro-inflammatory cytokines like IL-1β and IL-18, exacerbating inflammation and contributing to the breakdown of the blood-retinal barrier (33, 34). Our findings highlighted the involvement of PANoptosis-related genes in TNF and Toll-like receptor signaling pathways, underscoring their critical roles in mediating inflammation and retinal damage. Targeting these pathways could potentially mitigate inflammation and protect retinal cells. Similar observations have been reported in recent studies, underscoring the importance of these signaling pathways in DR pathogenesis. For instance, inflammation and VEGF-mediated pathways are crucial in DR and diabetic macular edema, with therapies targeting these pathways being essential for management (27). Inflammatory cells and cytokines contribute to micro-inflammation and the disruption of the blood-retinal barrier in DR (8). Additionally, multiple biochemical pathways activated during diabetes elevate the expression of angiogenic and inflammatory mediators, exacerbating retinal damage (35).

Through machine learning approaches including LASSO regression, RF algorithm, and SVM-RFE, we identified six key hub genes: BEX2, CASP2, CD36, FASN, OSMR, and PLSCR3. Notably, these hub genes have been previously associated with various cell death and inflammatory mechanisms, supporting their roles as pivotal regulators in DR. BEX2 is known for regulating cell survival and apoptosis, potentially protecting retinal neurons from hyperglycemia-induced damage in DR (36). CASP2, a critical enzyme in apoptosis, is linked to increased retinal cell death under diabetic conditions, contributing to neurodegeneration in DR (37, 38). CD36, a scavenger receptor involved in lipid metabolism and inflammation, is associated with oxidative stress and inflammation in DR (39, 40). FASN plays a significant role in lipid biosynthesis, with dysregulation contributing to retinal vascular abnormalities and inflammation in DR (41). PLSCR3, involved in mitochondrial function and apoptosis, is linked to mitochondrial dysfunction and cell death in retinal cells under hyperglycemic conditions (42). OSMR mediates inflammatory signaling pathways, with increased expression contributing to the chronic inflammation and vascular permeability observed in DR (43, 44).

To further evaluate the diagnostic value of these hub genes, we constructed a nomogram model. The nomogram is a predictive tool that integrates multiple predictors to estimate the probability of a clinical event (45). In our study, the nomogram was constructed by incorporating the six hub genes identified through machine learning. Calibration curves, DCA, and ROC curves were used to validate the model’s accuracy and clinical utility. The calibration curves indicated good agreement between predicted and observed outcomes (46), while the DCA demonstrated the clinical benefits of using the nomogram (47). The ROC curves further confirmed the high diagnostic accuracy of the nomogram, reinforcing the potential of these genes as robust biomarkers for DR (48).

GSVA further elucidated the association of these hub genes with crucial signaling pathways such as WNT/β-catenin signaling, Notch signaling, and apoptosis. The WNT pathway is known to play a critical role in regulating various physiological and pathological processes, including cell growth, apoptosis, and angiogenesis (49, 50). Recent studies have indicated that WNT signaling is activated in the retina of both human patients and animal models with DR, contributing significantly to the disease’s progression (51). Specifically, the activation of these pathways in DR suggests a complex regulatory network involving both inflammatory and cell death processes, with WNT signaling being particularly pivotal in this context (52).

The interplay between PANoptosis and mechanisms like autophagy, senescence, and ferroptosis adds complexity to DR pathogenesis. Autophagy, typically a survival process, can lead to cell death if dysregulated, affecting cell fate in DR (53). Senescence, marked by cell cycle arrest, may exacerbate retinal damage by promoting inflammation through the senescence-associated secretory phenotype (54, 55). Ferroptosis, an iron-dependent cell death, shares pathways with PANoptosis, particularly in oxidative stress regulation (56, 57). Understanding these intersections could reveal therapeutic targets to modulate cell death and inflammation in DR.

Immune cell infiltration analysis revealed distinct changes in immune cell populations in DR patients, particularly linking activated CD4 memory T cells, monocytes, and M0 macrophages with the expression levels of the hub genes. Activated CD4 memory T cells are known to play critical roles in autoimmune responses and chronic inflammation, which are key features in the pathogenesis of DR (58). Monocytes and M0 macrophages contribute to tissue damage by secreting pro-inflammatory cytokines and phagocytosing cellular debris (59). The expression levels of hub genes like CASP2 and CD36 were found to correlate with the abundance of these immune cell types, suggesting that these genes may influence immune cell behavior and contribute to disease progression (60, 61).

Based on our bioinformatics analysis of the dataset GSE221521, we identified six hub genes. Since this dataset was derived from peripheral blood samples, we initially collected peripheral blood samples from clinical patients with DR to validate our findings using RT-qPCR experiments. Firstly, RT-qPCR analyses of peripheral blood from DR patients indicated significant upregulation of the hub genes CASP2, CD36, and FASN, while BEX2 was significantly downregulated. Secondly, IHC staining was performed to visualize the protein expression of CD36, FASN, and PLSCR3 in the fibrovascular membranes of patients with PDR. The IHC results corroborated the RT-qPCR findings, confirming the upregulation of these genes in PDR tissues.

Finally, *in vitro* studies with HUVECs, a model widely recognized for its relevance and reproducibility in vascular research, exposed to hyperglycemic conditions, mimicked the diabetic environment and showed similar expression changes to those observed in patient samples, except for PLSCR3 (62). Alongside these studies, Western blot experiments were carried out to verify protein-level expression changes for CD36, FASN, and PLSCR3. These experiments reinforced previous research findings and revealed that while PLSCR3 mRNA levels exhibited no change, its protein expression was notably increased. This discrepancy highlighted the potential for post-transcriptional regulation mechanisms affecting PLSCR3. Moreover, the results for FASN and PLSCR3 demonstrated their ability to promote the proliferation and migration of HUVECs, suggesting a key role in vascular pathology associated with DR. In the context of PANoptosis, these findings emphasized the significance of FASN and PLSCR3 not only in endothelial cell behavior but also in their potential involvement in cell death pathways that integrate components of pyroptosis, apoptosis, and necroptosis. Specifically, the role of PANoptosis in vascular endothelial cells has been validated in various contexts, including renal injury induced by trichloroethylene, demonstrating HUVECs’ reliability for studying endothelial responses (63).

While our study provides valuable insights into the molecular mechanisms underlying diabetic retinopathy, several limitations should be acknowledged. Firstly, the relatively small sample size may limit the generalizability of our findings, necessitating validation in larger, more diverse cohorts. Additionally, while powerful, the use of advanced machine learning algorithms carries inherent risks of overfitting, which should be considered when interpreting our results. Although we validated the expression of hub genes through RT-qPCR and Western blotting, further *in vivo* and *in vitro* studies are necessary to confirm their functional roles in diabetic retinopathy. Understanding how these identified hub genes directly regulate PANoptosis in retinal cells remains a priority for future research. Addressing these limitations will be crucial for advancing our understanding and treatment of this condition.

## 5 Conclusion

In this study, we identified six pivotal hub genes (BEX2, CASP2, CD36, FASN, OSMR, and PLSCR3) associated with DR using advanced machine learning techniques. These genes were validated through multiple methods, including RT-qPCR, IHC, and Western blotting analyses, and further supported by bioinformatic validation. Our analyses, including GSVA and immune cell infiltration studies, highlighted the involvement of these genes in key signaling pathways and immune responses, particularly their roles in PANoptosis. Our findings suggest these genes hold promise as biomarkers and therapeutic targets, offering new insights into the molecular mechanisms of DR.

## Data Availability

The original contributions presented in the study are included in the article/**Supplementary Material**. Further inquiries can be directed to the corresponding author.
