# Supplementary figures and images for: Integrative analysis of PANoptosis-related genes in diabetic retinopathy: machine learning identification and experimental validation
Source: Front Immunol. 2024 Dec 4;15:1486251. doi: 10.3389/fimmu.2024.1486251 (PMC11652367; doi:10.3389/fimmu.2024.1486251)

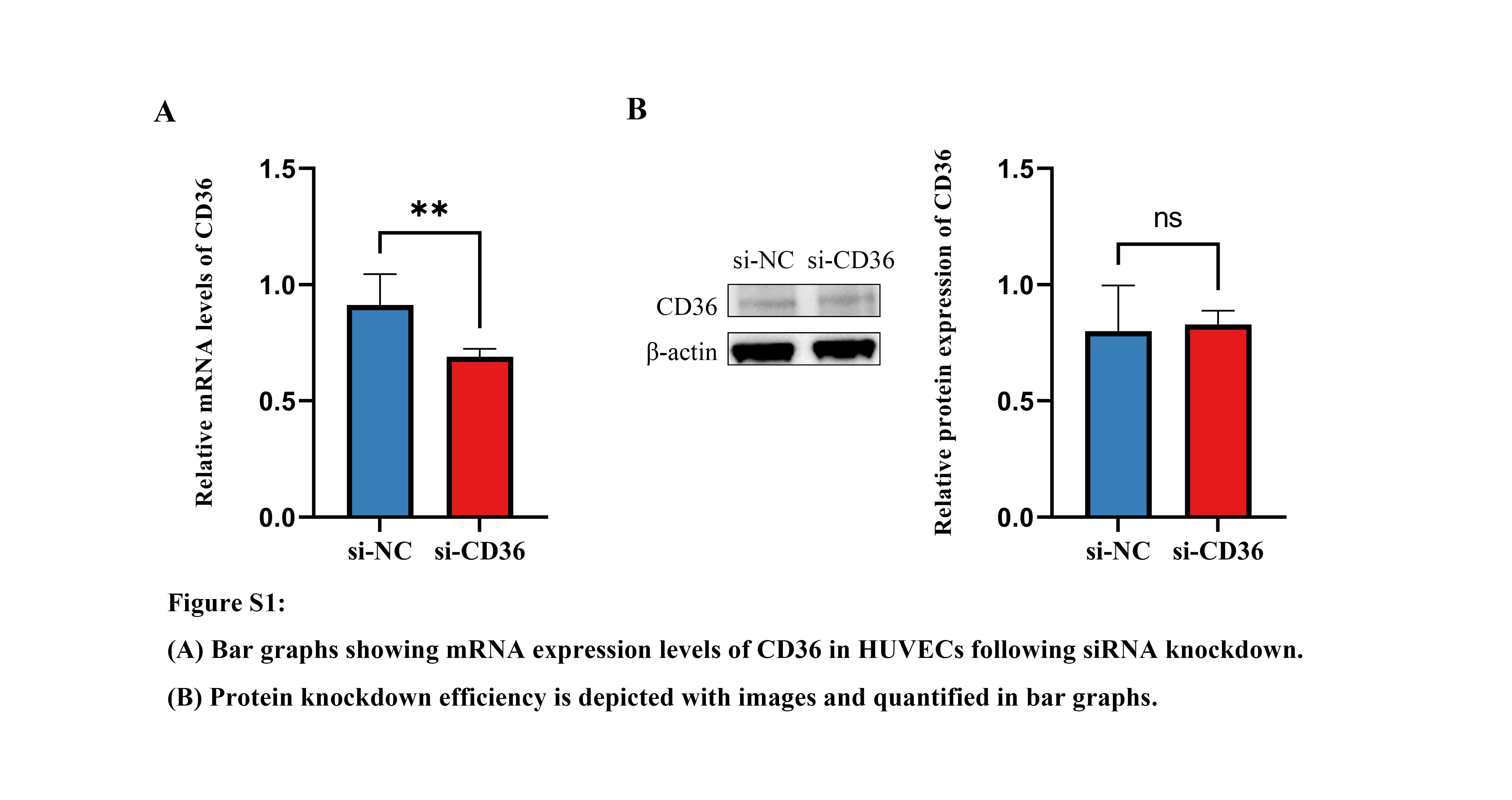

Supplement: Supplementary file 1 [file Image1.tif]
