# Supplementary material for: Integrative analysis of PANoptosis-related genes in diabetic retinopathy: machine learning identification and experimental validation
Source: Front Immunol. 2024 Dec 4;15:1486251. doi: 10.3389/fimmu.2024.1486251 (PMC11652367; doi:10.3389/fimmu.2024.1486251)
Supplement: Supplementary file 4 [file DataSheet1.docx]

**Supplemental Table S1. Quantitative PCR primers utilized in the research**.

| **Genes** | **Primer sequences (from 5' to 3')** |
| --- | --- |
| β-actin | F: 5’-CATGTACGTTGCTATCCAGGC-3’ |
|  | R: 5’-CTCCTTAATGTCACGCACGAT-3’ |
| BEX2 | F: 5’-TTGGAGAGCCACAGGCAAGGAT-3’ |
|  | R: 5’-AGTGCTGACTGCCCGCAAACTA-3’ |
| CD36 | F: 5’- GGCTGTGACCGGAACTGTG-3’ |
|  | R: 5’- AGGTCTCCAACTGGCATTAGAA-3’ |
| CASP2 | F: 5’- TGCCTTCTGTGAAGCACTGAGG-3’ |
|  | R: 5’- CGGAAAAGGGAGACTCAAGTCG-3’ |
| FASN | F: 5’- TTCTACGGCTCCACGCTCTTCC-3’ |
|  | R: 5’- GAAGAGTCTTCGTCAGCCAGGA-3’ |
| PLSCR3 | F: 5’- TGGATGTGAGGGTGAAGGCTGT-3’ |
|  | R: 5’- TGGAGTTCTGGTCGAGGTGATG-3’ |
| OSMR | F: 5’- CAGGTGTTCCTACCAAATCTGCG-3’ |
|  | R: 5’- AATCCACCCTCTGTGCCTGCAA-3’ |

**Supplemental Table S2. siRNA target sequences.**

| **Name** | **Sequences** |
| --- | --- |
| **Primers for real-time PCR:** |  |
| si-CD36 sense | 5'- GUCACUGCGACAUGAUUAATT -3' |
| si-CD36 antisense | 5'- UUAAUCAUGUCGCAGUGACTT -3' |
| si-FASN sense | 5'- GCCGAGUACAAUGUCAACATT-3′ |
| si-FASN antisense | 5'- UGUUGACAUUGUACUCGGCTT-3′ |
| si-PLSCR3 sense | 5'- CCUGUGAGCUGCCGAGUGCTT-3′ |
| si-PLSCR3 antisense | 5'- GCACUCGGCAGCUCACAGGTT-3′ |
| si-NC sense | 5'-UUCUCCGAACGUGUCACGUTT-3′ |
| si-NC antisense | 5'-ACGUGACACGUUCGGAGAATT-3′ |
